# Conformational Changes of rBTI from Buckwheat upon Binding to Trypsin: Implications for the Role of the P_8_′ Residue in the Potato Inhibitor I Family

**DOI:** 10.1371/journal.pone.0020950

**Published:** 2011-06-15

**Authors:** Longfei Wang, Fei Zhao, Mei Li, Hongmei Zhang, Yu Gao, Peng Cao, Xiaowei Pan, Zhuanhua Wang, Wenrui Chang

**Affiliations:** 1 National Laboratory of Biomacromolecules, Institute of Biophysics, Chinese Academy of Sciences, Beijing, People's Republic of China; 2 Graduate University of the Chinese Academy of Sciences, Beijing, People's Republic of China; 3 Key Laboratory of Chemical Biology and Molecular Engineering of the Ministry of Education, Institute of Biotechnology, Shanxi University, Taiyuan, People's Republic of China; Institute of Molecular and Cell Biology, Singapore

## Abstract

BWI-1 (buckwheat trypsin inhibitor), a member of the potato inhibitor I family, suppresses the growth of T-acute lymphoblastic leukemia cells and induces apoptosis in human solid tumor cell lines. Here, we report the crystal structure of rBTI (recombinant buckwheat trypsin inhibitor), a recombinant protein of BWI-1, at 1.84 Å resolution and the structure of rBTI in complex with bovine trypsin at 2.26 Å resolution. A conformational change of Trp53 at the P_8_′ position in rBTI was observed upon its binding to trypsin, which is not seen in other members of the potato inhibitor I family reported previously. The role of the P_8_′ residue in the potato inhibitor I family was examined by measuring the association and dissociation rates of four rBTI mutants with different substitutions at the P_2_ and P_8_′ positions when binding to trypsin. One of the mutants, P44T, was found to be a much stronger inhibitor than wild-type rBTI, with a picomolar (pM) dissociation constant. Our results could provide valuable insights for designing a new rBTI-based antitumor drug in the future.

## Introduction

Canonical inhibitors of serine protease function according to the standard mechanism of protease inhibition in which they bind tightly in the active site of a cognate protease in a substrate-like manner (substrate residues of protease inhibitors surrounding the cleavage site are designated by the nomenclature of Schechter and Berger [1]. The scissile bond is the starting point. In the direction of the N terminus, substrate residues are numbered P_1_, P_2_, P_3_ and so on, and in the direction of the C terminus, residues are numbered P_1_′, P_2_′, P_3_′ and so on.) [2]. However, unlike substrates, canonical inhibitors cannot be easily hydrolyzed by proteases, which is attributed to the rigidity of their convex binding loop [3]. The protein core of a canonical inhibitor serves as a scaffold for the binding loop and is responsible for maintaining the binding loop stability. A previous study revealed that an inhibitor could quickly form an acyl-enzyme intermediate with a protease but was hydrolyzed very slowly. Thus, a clogged gutter mechanism was proposed to underscore two key factors in protease inhibition: the intramolecular hydrogen-bonding network and the correct orientation of the religating amide [4].

The potato inhibitor I family belongs to the canonical inhibitors, and their P_2_, P_1_^′^, P_6_′, and P_8_′ residues are highly conserved due to their importance in the formation of the internal hydrogen-bonding network between the binding loop and protein core. Mutations of either P_2_ Thr or P_1_′ Glu in CI-2 (chymotrypsin inhibitor 2) result in a dramatic increase of the dissociation constant between CI-2 and chymotrypsin [5]. P_6_′ and P_8_′ mutants of CMTI-V (cucurbita maxima trypsin inhibitor V) have been proven to be very unstable. The P_6_′ mutant, in particular, can be easily hydrolyzed by trypsin [6].

Recently, attentions have been drawn to another member of the potato inhibitor I family from buckwheat seeds, BWI-1 (Buckwheat Inhibitor 1). BWI-1 was sequenced and characterized in buckwheat seeds soon after its discovery [7], [8], [9]. A previous cytobiology study revealed that BWI exhibits suppression activity against human T-Acute lymphoblastic leukemia cell lines [10]. In the past few years, Wang and her colleagues has focused on the antitumor activity of the BWI-1 recombinant protein rBTI (recombinant buckwheat trypsin inhibitor) [11] and has investigated its effects on the induction of apoptosis in several human solid tumor cell lines (EC907, HepG2 and HeLa) [12]. Additionally, the resistance of tobacco and potatoes to biotic stress can be improved by introducing the BWI-1 encoding gene [13].

Interestingly, BWI-1 has an uncommon binding loop sequence with a Pro at the P_2_ position and Trp at the P_8_′ position, suggesting a unique mode of intramolecular interactions between the binding loop and the protein core. Because the inhibition activity of certain canonical inhibitors is strongly affected by their intramolecular hydrogen-bonding network [4], it is logical to propose that BWI-1 inhibits proteases in an unusual way.

Here, we report the crystal structure of rBTI at 1.84 Å resolution and the structure of rBTI-trypsin complex at 2.26 Å resolution. Curiously, structural superposition revealed a significant conformational change of P_8_′ Trp in rBTI upon binding to trypsin. Several rBTI mutants were constructed to mimic different binding loop conformations of potato inhibitor I family members. Their association and dissociation rates upon binding to bovine trypsin were determined, allowing us to correlate several binding loop conformations with their inhibition abilities in the potato inhibitor I family. Out of our expectations, one of the mutants, P44T, was found to be a much stronger inhibitor compared to the wild-type with a picomolar (pM) dissociation constant. These results allow us to propose a detailed model for the structural basis of protease inhibition of the potato Inhibitor I family.

## Results and Discussion

### Overall structure of native rBTI and its complex with bovine trypsin

**Figure 1:**
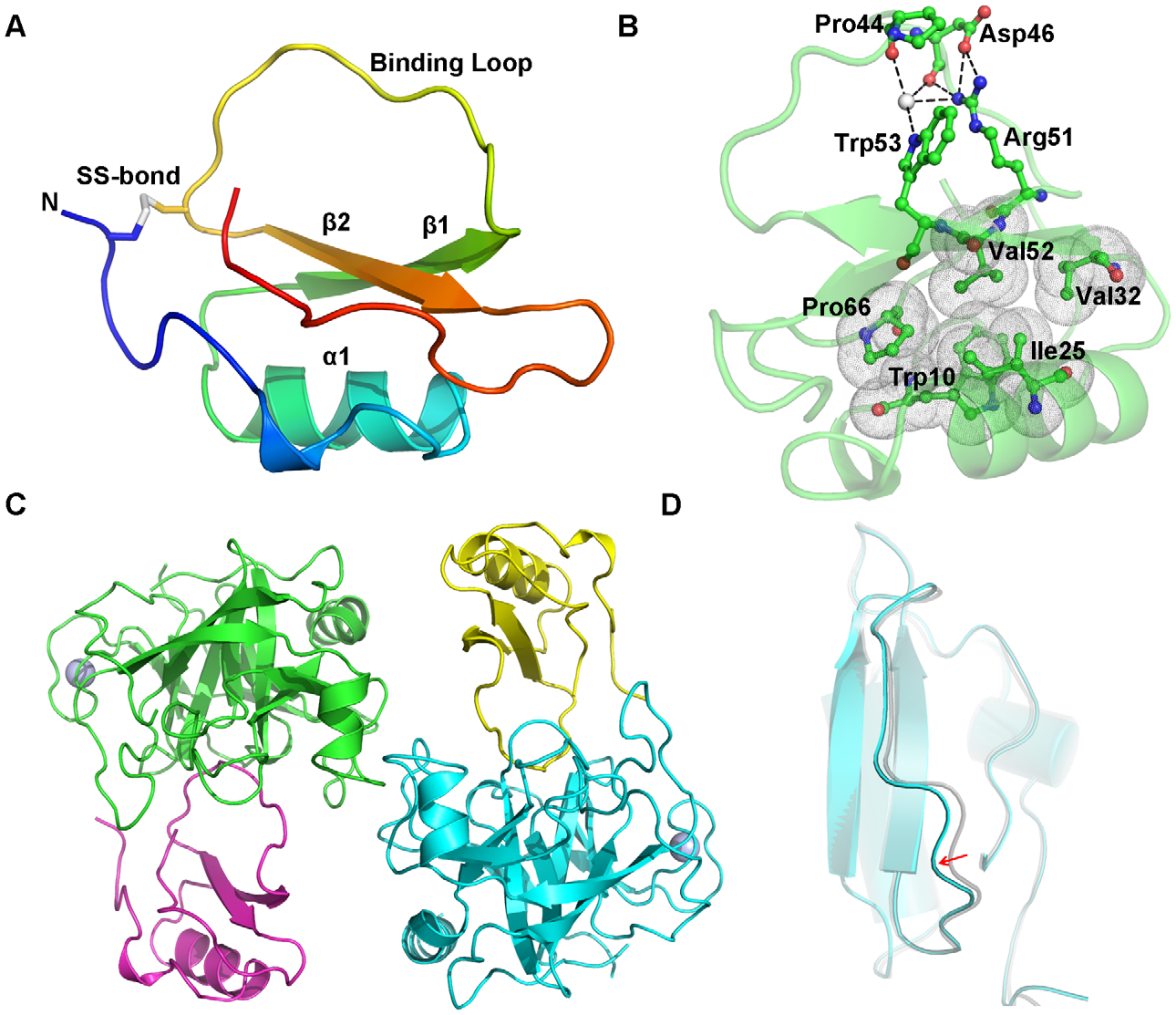
(A) Cartoon representation of the overall structure of rBTI. Different structural elements are shown in different colors, and the disulfide bridge is indicated. (B) A view of the hydrogen-bonding network and the hydrophobic core in rBTI. rBTI is shown in a cartoon presentation in green. Residues involved in hydrogen-bonding network and hydrophobic core are shown as ball-and-stick models. The grey sphere indicates a water molecule. Hydrogen bonds are indicated by black dashes and hydrophobic interactions are indicated by dotted clouds. (C) Overview of the structure of rBTI-trypsin complexes within an asymmetric unit. rBTIs are shown in yellow and magenta; trypsins are shown in green and cyan. The calcium ions in trypsin are shown as light-blue spheres. (D) Superposition of trypsin-bound rBTI and free rBTI. The binding loop of trypsin-bound rBTI (cyan) is shifted by a small distance from that of free rBTI (grey). The RMSD value calculated by superposition of trypsin-bound rBTI's and free rBTI's binding loops is 0.26 Å.

The structure of rBTI is composed of 69 amino acid residues. Its main structural elements comprise a single α-helix (α1, residues 18 to 28), a central parallel β-sheet consisting of two strands (β1, residues 30 to 38; β2, residues 51 to 56), a binding loop (residues 39 to 50) and two irregular structures at the N-terminus (residues 3 to 15) and C-terminus (residues 61 to 69)(Figure 1A). A hydrophobic core is formed in rBTI among α1, β1, β2 and two short loops (side-chains of Trp10, Ile25, Val32, Val52 and Pro66), as shown in Figure 1B. The Cys4-Cys49 disulfide bond stabilizes the binding loop by connecting it with the N-terminus. The binding loop of rBTI is a convex loop sandwiched between β1 and β2. Within the loop, the P_1_ residue, Arg45, is an ideal substrate of trypsin, which ensures the inhibitor's tight binding to trypsin. As in other canonical inhibitors, the binding loop of rBTI forms a hydrogen-bonding network with the protein core [6], [14], [15](Figure 1B). This hydrogen-bonding network is one of the key factors that causes inhibitors to be hydrolyzed at a very slow rate [3].

In the crystal structure of the rBTI-trypsin complex, one crystallographic asymmetric unit contains two rBTI-trypsin complexes, that is, two rBTIs and two trypsins, as shown in Figure 1C. The structure of bovine trypsin in complex with rBTI aligns well with other trypsin structures deposited in the PDB database. Trypsin-bound rBTI has an overall structure similar to that of free rBTI, with both consisting of one α-helix and a central parallel β-sheet. By superposing trypsin-bound rBTI over free rBTI, we found that the Arg45 at the P_1_ position was buried deeply into the binding pocket of trypsin, leading to a small but noticeable shift of the binding loop towards trypsin (RMSD 0.26 Å, Figure 1D). This movement disrupts several hydrogen bonds between the binding loop and protein core, indicating that a significant conformational change of the binding loop occurs upon binding to trypsin.

### Comparison of the binding loops between rBTI and LUTI

As noted, the P_6_′ and P_8_′ residues play important roles in maintaining the stability of the binding loops of inhibitors in the potato inhibitor I family. Mutations of the P_6_′ and P_8_′ residues would destabilize the binding loop structure, resulting in a significant decrease in the inhibitor's activity [6], [15]. Therefore, the P_6_′ and P_8_′ residues are highly conserved among members of the potato inhibitor I family. Almost all reported structures in the potato inhibitor I family exhibit an Arg at the P_8_′ position, with the exception of rBTI and LUTI (linum usitatissimum trypsin inhibitor), in which the P_8_′ residue is Trp (Figure S1). It is noteworthy that although the sequence homology of LUTI and rBTI is very high (58% sequence identity), their binding loops have completely different conformations.

**Figure 2:**
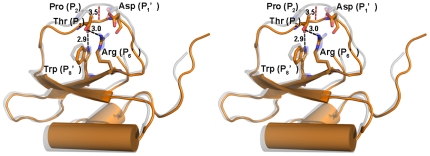
Stereo view of the superposition of LUTI and rBTI showing the different conformations of their binding loops. LUTI is shown in brown, and rBTI is shown in grey. Red dashes indicate the distance between the Cα atoms of the P_1_ residues in rBTI and LUTI.

In the structure of LUTI, Thr44 at the P_2_ position forms hydrogen bonds with Arg51 at the P_6_′ position and Trp53 at the P_8_′ position (Figure 2). Cierpicki *et al.* proposed that because Trp has a shorter side-chain than Arg, the binding loop of LUTI would be much closer to the protein core than other members of the potato inhibitor I family [14].

However, in the case of rBTI, there is a Pro at the P_2_ position instead of Thr. Therefore, no hydrogen bond can be formed between the P_2_ and P_8_′ residues or between the P_2_ and P_6_′ residues, resulting in an extended binding loop distant from the protein core. After superposing LUTI over rBTI, we observed that the binding loop of rBTI was approximately 3.5 Å more distant from the protein core than that of LUTI. Moreover, the P_2_ Pro appears to be responsible for the conformational change of the binding loop after rBTI binds to trypsin.

### Conformational change of the binding loop of rBTI

Given that rBTI presents an uncommon residues (Trp) at the P_8_′ position and that its binding loop is different from that of LUTI, which also has a Trp at the P_8_′ position, further investigation of the P_8_′ Trp of rBTI appears to be a promising means through which to shed some light on the inhibition mechanism of rBTI.

**Figure 3:**
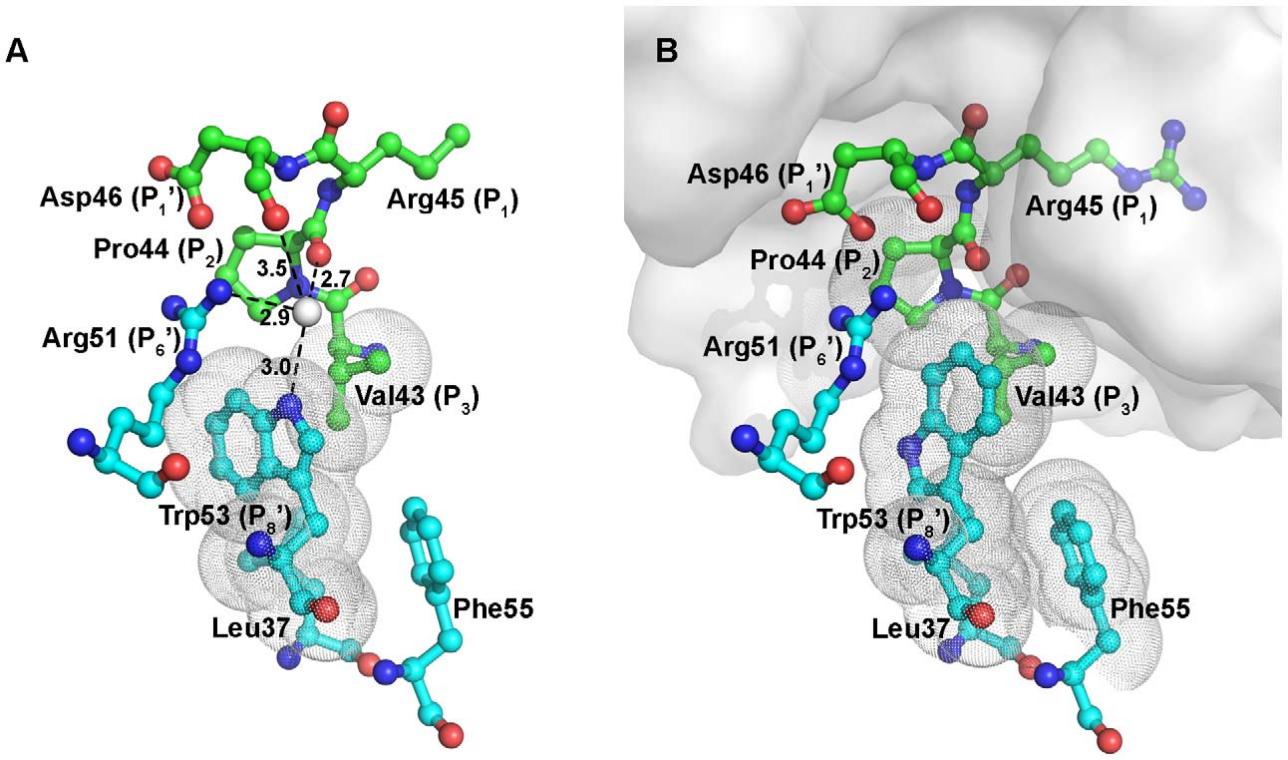
Structural differences between free rBTI and trypsin-bound rBTI at the local region around the P_8_′ position. (A) Interactions between the P_2_ and P_8_′ residues in free rBTI. (B) Interactions between the P_2_ and P_8_′ residues in trypsin-bound rBTI. Side-chains of residues in the binding loop are shown in green, while those in the protein core are shown in cyan. The grey sphere indicates a water molecule. Trypsin is shown as surface. Hydrophobic interactions are indicated by dotted clouds. Part of the side chain of Arg45 in free rBTI is missing due to poor electron densities.

It is generally believed that the P_8_′ residue, as well as other residues that form the intramolecular hydrogen-bonding network in the potato inhibitor I family, maintains a relatively stable conformation upon binding to proteases [3]. However, superposition of free rBTI and trypsin-bound rBTI suggests that a significant conformational change occurs when rBTI binds to trypsin. As shown in Figure 3, in free rBTI, a water molecule forms hydrogen bonds with Trp53 at the P_8_′ position, Arg51 at the P_6_′ position, the α carbonyl oxygen of Pro44 at the P_2_ position and the α carbonyl oxygen of Asp46 at the P_1_′ position. This water molecule mediates the interactions between the binding loop and the protein core, stabilizing the structure of the binding loop. When rBTI binds to trypsin, its P_1_ residue fits into trypsin's binding pocket and forms hydrogen bonds with trypsin residues. Because of this tight binding, the binding loop of rBTI is pulled towards trypsin slightly (Figure 1D), which shifts the P_2_ and P_1_′ residues further from the protein core and causes a rupture of the hydrogen bonds around the water molecule, leading to the dissociation of this water molecule. As a consequence, the binding loop of rBTI cannot maintain a stable conformation and risks being hydrolyzed by trypsin, similarly to a regular substrate. To counteract with this situation, rBTI employs a local conformational change mechanism: the Trp53 at the P_8_′ position undergoes a rotameric switch, with its indole ring flipping upwards and occupying the original position of the water molecule (Figure S3). In this new conformation, Trp53 forms a stable cation-π interaction (examined by the CaPTURE program [16]) with Arg51 at the P_6_′ site and interacts with hydrophobic residues from the binding loop, thereby stabilizing the binding loop.

### Binding loop conformations of the potato inhibitor I family and their inhibition activities

Following the discovery of the rotameric switch of the P_8_′ Trp in rBTI, two questions arose: why does rBTI exhibit such a rare inhibition mechanism; and what roles do the P_8_′ and P_2_ residues play in its inhibition activity? To address these questions, we referred to the structure of several classical members of the potato inhibitor I family and designed four rBTI mutants. These mutants each include substitutions at the P_2_ and P_8_′ positions to mimic different binding loop conformations of the potato inhibitor I family members. Interactions of wild-type rBTI and rBTI mutants with bovine trypsin were investigated by means of an optical biosensor using the surface plasmon resonance (SPR) effect. Their association rate (k_a_) and dissociation rate (k_d_) and the dissociation constant (K_D_) were determined (Table 1, Figure S2).

**Table 1:** Association rates (k_a_), dissociation rates (k_d_) and dissociation constants (K_D_) for the interactions of wild-type rBTI and its mutants with bovine trypsin.

Inhibitor	P_8_′ residue	P_2_ residue	Speculated interaction	Reference Model	k_a_, M^−1^s^−1^	k_d_, s^−1^	K_D_(k_d_/k_a_), M
WT rBTI	Trp53	Pro44	Hydrophobic force	rBTI	4.62×10^5^	1.25×10^−3^	2.69×10^−9^
P44T	Trp53	Thr44	Hydrogen bond	LUTI	3.46×10^5^	5.24×10^−7^	1.52×10^−12^
W53R/P44T	Arg53	Thr44	Hydrogen bond	CI-2	6.79×10^5^	5.54×10^−4^	8.15×10^−10^
W53F	Phe53	Pro44	Hydrophobic force	-	3.99×10^5^	2.46×10^−3^	6.16×10^−9^
W53R	Arg53	Pro44	-	-	6.59×10^5^	9.97×10^−3^	1.51×10^−8^

**Figure 4:**
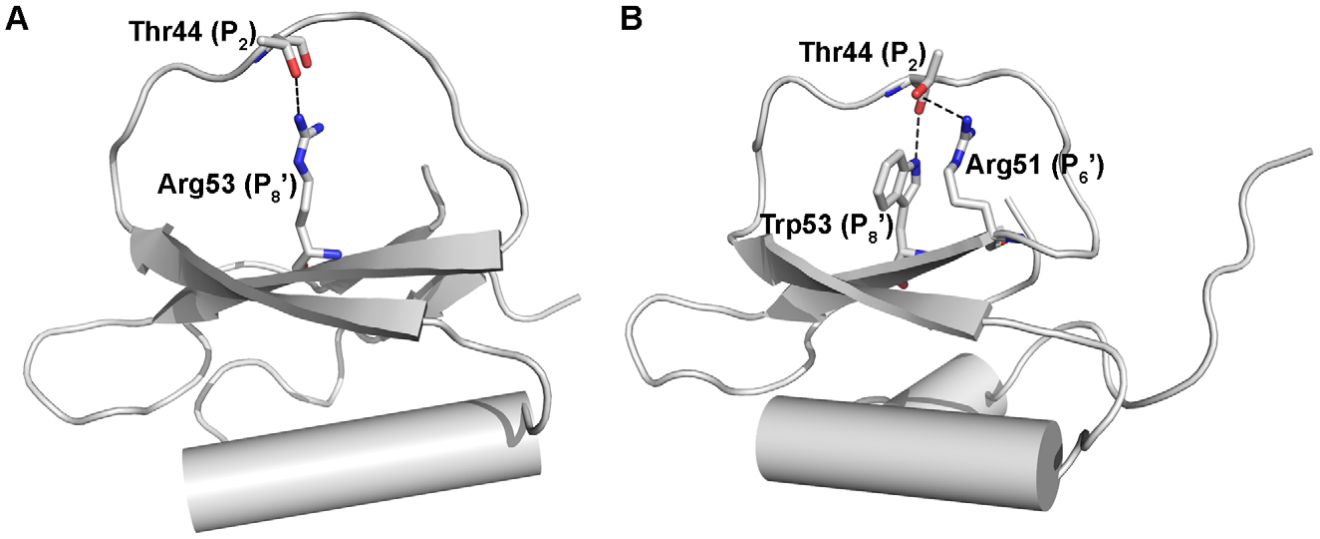
Putative rBTI mutant structures based on inhibitors homologous to rBTI. (A) Possible structure of W53R/P44T based on CI-2. (B) Possible structure of P44T based on LUTI.

Both rBTI mutants with an Arg substitution at the P_8_′ position (W53R, W53R/P44T) show elevated association rates compared to the wild-type rBTI for tryspin. As proposed previously, the closer packing of the binding loop of LUTI against the protein core compared to CI-2 and eglin C is attributed to the shorter side-chain of Trp compared to Arg [14]. We speculated that it is the same in rBTI: because Arg has a longer side-chain than Trp or Phe, mutants with Arg at the P_8_′ position (W53R, W53R/P44T) exhibit more extended binding loops than other forms of rBTI (wild-type rBTI, P44T, W53F) (Figure 4A). Thus, the binding loops of W53R and W53R/P44T are more accessible to the substrate pocket of trypsin, resulting in elevated association rates. As noted earlier, for wild-type rBTI, a water molecule mediates the interaction between the binding loop and protein core, suggesting that the binding loop of wild-type rBTI is also extended. This could explain why rBTI exhibits a third higher association rate among the wild-type rBTI and all rBTI mutants.

We also found that mutants that are able to form hydrogen bonds between the P_2_ and P_8_′ residues (P44T, W53R/P44T) present significantly lower dissociation rates compared to wild-type for trypsin. The most surprising outcome from our kinetic analyses is that the dissociation rate of P44T is three orders of magnitude lower than that of W53R/P44T, indicating that P44T is a very strong inhibitor to trypsin. We speculated that the much lower dissociation rate of P44T might be attributed to two factors: one is that a second hydrogen bond formed between P_2_ and P_6_′ (prediction based on the structure of LUTI, Figure 4B); the other is that the side-chain of Trp is more rigid than that of Arg. A combination of these two factors stabilizes the binding loop conformation, resulting in a more than 10^3^-fold decrease in the dissociation constant when it binds trypsin.

Among all investigated forms of rBTI, W53R exhibited the highest dissociation rate. In BIAcore binding assays, W53R was the only mutant that did not require the regeneration of immobilized trypsin, suggesting that it had lost the normal function of an inhibitor. The reason might be the lack of stable interactions formed between its P_2_ Pro and P_8_′ Arg. Therefore, the binding loop of W53R is very unstable and is vulnerable to hydrolysis by trypsin. This result underpins the important role of the P_8_′ residue in the inhibitory functioning of the potato inhibitor I family. However, our speculation needs further experimental proof.

Of all the inhibitors that cannot form a hydrogen bond between P_2_ and P_8_′, wild-type rBTI showed the lowest dissociation rate. We assumed that this was related to the conformational change within rBTI: upon binding to trypsin, Trp53 flips up and forms a relatively more stable conformation. In comparison with W53F, we found that wild-type rBTI had a slightly higher association rate and a lower dissociation rate, suggesting that the rotameric switch of Trp53 might improve the inhibition activity of wild-type rBTI.

Based on these results, we inferred that in the potato inhibitor I family, at least, the association rate and dissociation rate of an inhibitor to a protease are not necessarily related, which is consistent with the results of a previous study [17]. In our study, we found that the association rate was determined by the extended shape of the binding loop, as well as its amino acid composition [18]. However, the dissociation rate is impacted by the overall structure of the inhibitor, in particular, the internal hydrogen-bonding network between the binding loop and the protein core. Based on the analysis of our data, we further speculated that in the potato inhibitor I family, inhibitors with more extended binding loops bind to protease at a higher association rate; interactions between the P_8_′ and P_2_ residues of an inhibitor can significantly affect the dissociation rate to its cognate protease, which can be summarized as: k_d-hydrogen_<k_d-nonhydrogen_<k_d-non_ in which k_d-hydrogen_ is the dissociation rate of inhibitors that have a hydrogen bond between the P_8_′ and P_2_ residues, while k_d-nonhydrogen_ denotes the dissociation rate of inhibitors presenting interactions other than hydrogen-bonding between the P_8_′ and P_2_ residues, and k_d-non_ denotes inhibitors exhibiting no interaction between the P_8_′ and P_2_ residues. Among inhibitors that form hydrogen bonds between P_8_′ and P_2_, those that can form extra hydrogen bonds or present a more rigid P_8_′ side-chain exhibit the lowest dissociation rate. Our conclusion might also apply to other types of canonical inhibitors that form hydrogen bonds at different positions.

Research on the potato inhibitor I family began in the mid-19th century. However, conformational changes of the P_8_′ residues of these inhibitors upon binding to proteases in a similar way to what is seen for rBTI have not been reported before. In the present study, we compared several different binding loop conformations in the potato inhibitor I family on the same protein core. Such comparisons allowed us to better understand the relationship between the conformation of the binding loops and their inhibition activities.

This comparison also provided us a clearer picture of the inhibition mechanism of rBTI: the rotameric switch of the P_8_′ Trp results in rBTI being a relatively weak inhibitor but does not compromise its proteolytic stability, which is a reflection of biological diversity. In this case, rBTI could be suitable for certain physiological processes that require a weak, but stable, inhibitor.

It is also worth mentioning that the P44T mutant we constructed presents a dissociation constant three orders of magnitude lower than that of wild-type rBTI. This mutant may represent a good example of improving an inhibitor's binding affinity to its cognate protease. Therefore, it could provide useful information for improving the binding affinity of inhibitor drugs to their target proteins.

As noted earlier, although rBTI can suppress tumor cell growth and induce apoptosis in several tumor cell lines [10], [11], [12], the mechanism underlying its antitumor activity is currently unknown. This structural study of rBTI represents a first step towards understanding its antitumor mechanism. Moreover, The rBTI mutants with an improved inhibition activity for trypsin investigated in the present study may facilitate designing inhibitors with higher antitumor activities and are of potential therapeutic value.

## Materials and Methods

### Expression, purification and crystallization of rBTI and the rBTI-trypsin complex

rBTI was prepared as described previously [11]. The rBTI crude sample was then applied to Superdex75 (GE Healthcare). The elution buffer used was 25 mM Tris-HCl (pH 8.0) and 50 mM NaCl. The fractions containing rBTI were collected and concentrated to 20 mg/ml. rBTI was crystallized by vapor diffusion. Crystals were grown at 18°C in hanging drops over a reservoir of 24% (w/v) PEG MME2000, 220 mM (NH_4_)_2_SO_4_, 100 mM NaAc (pH 4.4) and 100 mM NaI. Drops were prepared by mixing equal volumes of protein and reservoir solutions. After two weeks, thin and rod-like crystals were harvested, soaked in a cryoprotectant mixture (paraffin oil and NVH oil in a ratio of 7∶3) and flash-frozen in liquid nitrogen.

rBTI and bovine trypsin (AppliChem) were mixed in a 1∶1.3 stoichiometric molar ratio and incubated at room temperature for a half an hour to form complexes. The incubation buffer contained 50 mM Tris-HCl (pH 8.0) and 200 mM NaCl. After incubation, the incubation sample was applied to Superdex75 to remove excessive rBTI. The elution buffer used was 50 mM Tris pH 8.0 and 20 mM NaCl. The complex of rBTI with bovine trypsin was also crystallized by vapor diffusion. Crystals were grown at 18°C in hanging drops over a reservoir of 15% (w/v) PEG3350, 200 mM MgCl_2_ and 100 mM Tris-HCl (pH 9.0). Drops were prepared by mixing equal volumes of protein and reservoir solutions. Rod-like crystals grew over the course of two weeks. They were then harvested, soaked in a cryoprotectant solution (100 mM Tris-HCl pH 9.0, 20% (w/v) PEG3350, 20% (v/v) glycerol and 200 mM MgCl_2_) and flash-frozen in liquid nitrogen.

### X-ray data collection and processing

For rBTI, synchrotron X-ray data were collected from a single crystal at 100 K using a MAR555 CCD detector at beamline 1W2B, BSRF (Beijing Synchrotron Radiation Facility). For rBTI in complex with bovine trypsin, X-ray data were collected from a single crystal at 100 K using a Raxis4 IP detector at the Institute of Microbiology, Chinese Academy of Science. All data were processed and scaled with the HKL2000 software suite [19].

### Phasing

The rBTI-trypsin complex was the first to yield diffraction quality crystals. An incomplete structure containing only trypsin was solved by molecular replacement using the program Phaser [20]. The search model was derived from a previous structure of trypsin (PDB entry 2CMY). After failed attempts at molecular replacement using two search models (LUTI, linum usitatissimum trypsin inhibitor, PDB entry 1DWM; CMTI-V, cucurbita maxima trypsin inhibitor-V, PDB entry 1 HYM) that share the highest sequence homology with rBTI, we used MrBump [21] to perform a search for homologous structures. Then, one of the two rBTIs in the asymmetric unit was solved using a search model of BGTI (Bitter Gourd Trypsin Inhibitor, PDB entry 1VBW). The other rBTI was solved by superposing one rBTI-trypsin complex over the other trypsin using trypsin as the reference structure in the asymmetric unit.

The rBTI structure was solved by molecular replacement using the program Phaser [20] with the solved trypsin-bound rBTI structure as a search model.

### Model Refinement

Models were rebuilt using the model-building module of the PHENIX software suite [22]. Cycles of manual rebuilding in COOT [23] were alternated with automated refinement using the refinement module of PHENIX. Test sets comprised 5% of the total reflections were excluded from refinement to allow the calculation of the free R-factors. Composite omit maps generated by the CNS software suite [24] and prime and switch maps generated by Resolve [25] were used as reference maps in manual rebuilding. Model validations were carried out using PROCHECK [26]. Superpositions were performed using COOT, andall figures representing structures were created using the graphics software PyMOL. A summary of the data collection and refinement statistics is presented in Table 2. The coordinates and structure factors of rBTI were deposited into RCSB Protein Data Bank with accession code 3RDY. The coordinates and structure factors of rBTI-trypsin complex were deposited with accession code 3RDZ.

**Table 2:** Summary of Data Collection and Refinement Statistics.

	rBTI-trypsin complex	rBTI
Wavelength (Å)	1.5418	1.00
Space group	P2_1_	P4_3_2_1_2
Resolution range^a^ (Å)	12.0−2.26(2.34−2.26)	15.0−1.84(1.91−1.84)
Unique Reflections	26279	8344
Unit Cell (a,b,c) (Å)	66.7, 50.2, 84.5	62.7,62.7,45.9
Completeness^a^ (%)	99.8(97.7)	99.8(100)
Redundency^a^	3.7(3.6)	24.1(23.2)
Average^a^ ^b^ I/σ	22.6(4.9)	46.4(4.7)
R_merge_ a (%)	5.5(25.3)	8.0(50)
a.s.u content		
No. trypsin	2	-
No. rBTI	2	1
No. Non-hydrogen atoms	4247	494
No. Ca^2+^	2	-
No. water molecules	300	94
R factor and R_free_ (%)	18.2/22.6	19.1/21.6
r.m.s deviations:		
Bond length (Å)	0.0075	0.012
Bond angles (deg)	1.116	1.390
B-factors (Å^2^):		
Protein	30.9	28.8
Main-chain	29.5	25.7
Side-chain and water	32.4	31.3

a Outer shell values are given in parentheses.

b I is the intensity; σ is the standard deviation.

### Expression and purification of the mutants

The rBTI expression construct was mutagenized using the PCR-based QuickChange method (Stratagene). We designed four mutants based on the structure of the homologous inhibitors of rBTI. They were P44T, W53F, W53R and W53R/P44T double mutant. The mutants of rBTI were expressed and purified in the same way as wild-type rBTI.

### BIAcore Binding Assays

Interactions of wild-type rBTI and its mutants with bovine trypsin (AppliChem) were measured using the optical biosensor BIAcore 3000 and CM5 optical chips. Carboxymethlated dextran on the chip surface was activated with the mixture 0.2 M EDC/0.05 M NHS. The subsequent immobilization of bovine trypsin was carried out by injecting a trypsin solution (20 µg/ml in 10 mM acetate buffer, pH 5.5) at a flow rate of 5 µl/min over the activated sensor surface. The residual active groups of dextran were blocked by 1 M ethanolamine.

Interactions of different inhibitors with immobilized trypsin were studied using concentrations of 7.4 nM, 22.2 nM, 66.7 nM and 200 nM (at a flow rate of 30 µl/min for 1 min) in running buffer containing 100 mM NaCl, 10 mM Na_2_HPO4∶NaH_2_PO_4_ (pH 8.0) (Figure S2). After the injection of each inhibitor sample, except W53R, the chip was regenerated by the injection of 10 mM Glycine-HCl (pH 3.0). A channel without immobilized protein was used as a reference. Running buffers without inhibitors were used to generate the baseline. Kinetic parameters were calculated using the program BIAevaluation, and the mathematical model was 1∶1 Langmuir binding.

## Supporting Information

Figure S1 **Sequence alignment of several members of the potato inhibitor I family.** The binding loop are marked with grey alpha boxes.
(TIF)

Figure S2 **Sensograms of the interaction of wild-type rBTI and rBTI mutants with immobilized bovine trypsin.** (A) Wild-type rBTI. (B) W53R/P44T double mutant. (C) W53F. (D) P44T. (E) W53R.
(TIF)

Figure S3 (A and B) P_8_′ Trp omit maps of free rBTI (A) and trypsin-bound rBTI (B). The 2Fo-Fc map is shown in blue, and the Fo-Fc map is shown in green and red. The positive electron density is shown in green, and the negative density is shown in red. (C and D) 2Fo-Fc (blue) and Fo-Fc (red and green) maps of P_8_′ Trp with incorrect conformations in free rBTI (C) and trypsin-bound rBTI (D). The positive electron density is shown in green, and the negative density is shown in red.
(TIF)
