# Supplementary figures and images for: Conformational Changes of rBTI from Buckwheat upon Binding to Trypsin: Implications for the Role of the P8′ Residue in the Potato Inhibitor I Family
Source: PLoS One. 2011 Jun 15;6(6):e20950. doi: 10.1371/journal.pone.0020950 (PMC3115953; doi:10.1371/journal.pone.0020950)

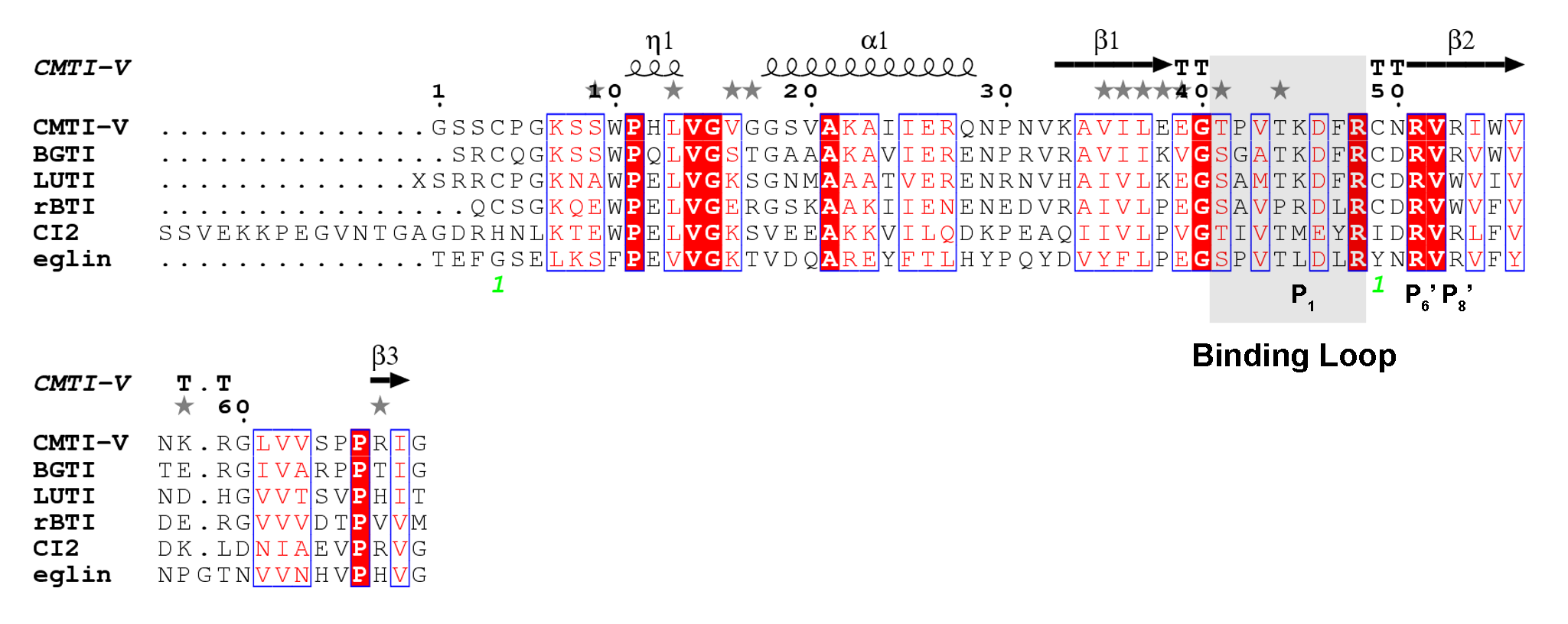

Supplement: Figure S1 — Sequence alignment of several members of the potato inhibitor I family. The binding loop are marked with grey alpha boxes. (TIF) [file pone.0020950.s001.tif]

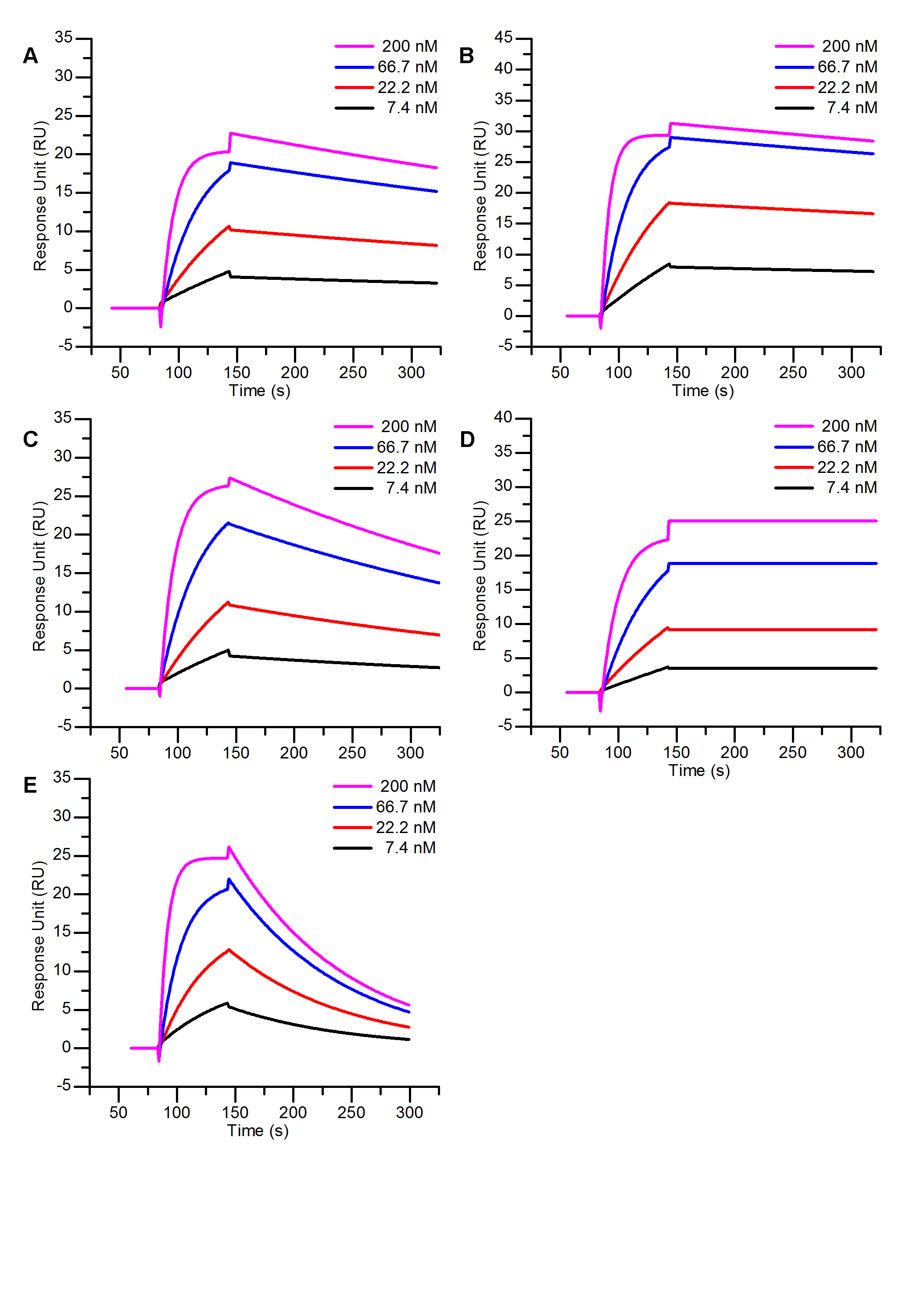

Supplement: Figure S2 — Sensograms of the interaction of wild-type rBTI and rBTI mutants with immobilized bovine trypsin. (A) Wild-type rBTI. (B) W53R/P44T double mutant. (C) W53F. (D) P44T. (E) W53R. (TIF) [file pone.0020950.s002.tif]

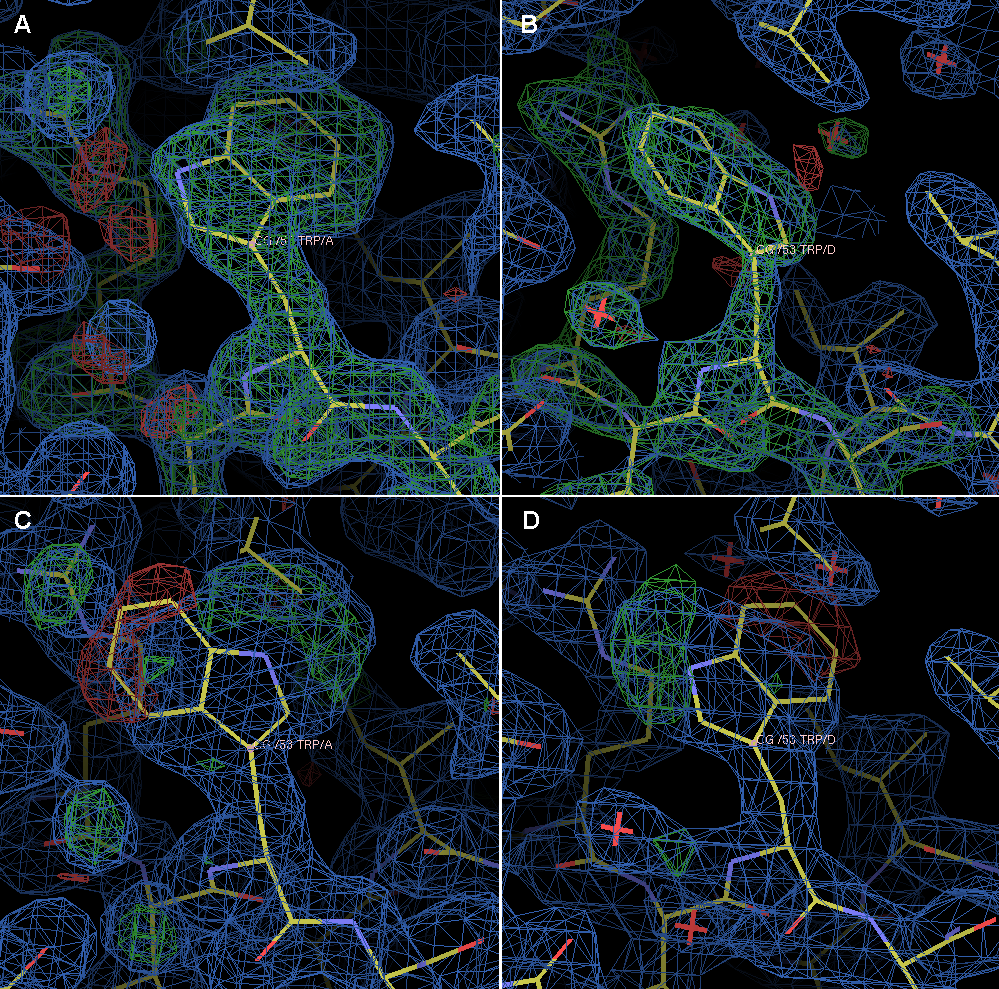

Supplement: Figure S3 — (A and B) P8′ Trp omit maps of free rBTI (A) and trypsin-bound rBTI (B). The 2Fo-Fc map is shown in blue, and the Fo-Fc map is shown in green and red. The positive electron density is shown in green, and the negative density is shown in red. (C and D) 2Fo-Fc (blue) and Fo-Fc (red and green) maps of P8′ Trp with incorrect conformations in free rBTI (C) and trypsin-bound rBTI (D). The positive electron density is shown in green, and the negative density is shown in red. (TIF) [file pone.0020950.s003.tif]
